# Ultrasound-Guided Erector Spinae Block for Refractory Abdominal Pain Due to Acute on Chronic Pancreatitis

**DOI:** 10.7759/cureus.31817

**Published:** 2022-11-23

**Authors:** Gaurav Chauhan, Harold Burke, Suresh K Srinivasan, Aman Upadhyay

**Affiliations:** 1 Anesthesiology and Perioperative Medicine, University of Pittsburgh Medical Center Presbyterian, Pittsburgh, USA; 2 Pain Management, Trinity West Medical Center, Steubenville, USA; 3 Pain Medicine, McLaren Oakland Hospital, Pontiac, USA

**Keywords:** abdominal pain, acute pancreatitis, chronic pain, regional anesthesia, erector spinal block

## Abstract

Erector spinae blocks (ESBs) are a type of fascial pain block that has been safely used for various applications, including post-operative and post-trauma pain in several thoracic and abdominal surgeries. Pain related to an acute flare-up of chronic pancreatitis is usually challenging to control and impacts patient comfort and discharge planning. This case report describes an application of ESBs for the effective treatment of refractory abdominal pain associated with acute exacerbation of chronic pancreatitis. Application of ESB in the emergency room setting can potentially decrease hospital admission for this common condition and increase patient satisfaction.

## Introduction

Acute pancreatitis is responsible for more than 200,000 hospital admissions every year, and this is the most common gastrointestinal disease causing a hospital admission [1]. Therefore, acute pancreatitis has a heavy burden on patient morbidity, mortality, and healthcare costs. In addition to bowel rest and hydration, pain management is an integral part of treatment. In addition, pain control is a limiting factor for discharge planning. Noxious stimuli from tissue injury and inflammation result in the transmission of pain signals to the spinal cord, although the exact mechanisms are unknown. Common options for medication management include non-steroidal anti-inflammatory medications, acetaminophen, and opioids, but their utility and efficacy are limited by patient comorbidities and the side effect profile.

Different types of fascial pain blocks have been described in recent years for a variety of clinical applications. These techniques can be easily performed in most clinical settings without extensive resources and a low-risk profile. With this case report, we would like to add evidence to the current body of literature supporting the utility of erector spinae blocks (ESBs) in managing severe abdominal pain associated with acute pancreatitis.

## Case presentation

**Figure 1:**
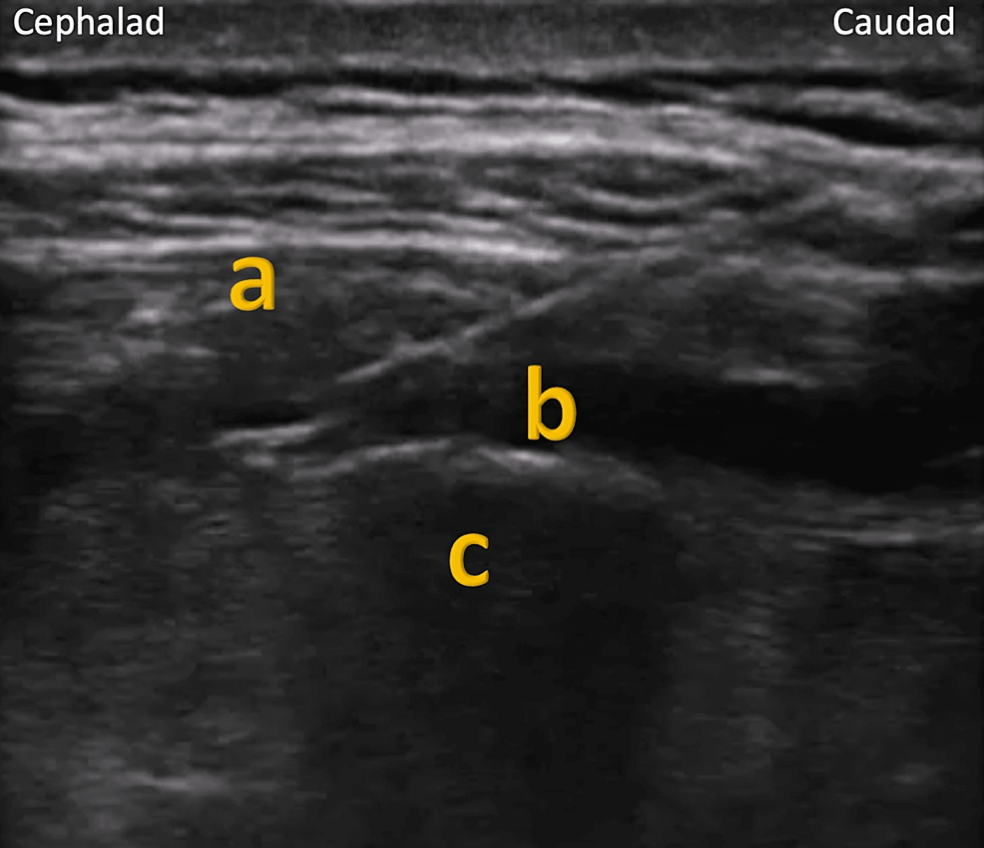
Ultrasonographic image of erector spine block with a mixture of local anesthetic and steroid below the erector spinae muscle at transverse process of T6 vertebrae. The image show (a) erector spinal muscle, (b) spread of local anesthetic and block solution below the erector spinal muscle, and (c) transverse process of T6 vertebrae.

A 61-year-old female patient with a past medical history of diabetes and pancreatitis presented to the hospital with three days of abdominal pain. The acute abdominal pain was attributed to an acute on chronic pancreatitis flare based on clinical findings and admission labs. The patient was experiencing significant aching and stabbing pain in the right and left upper quadrants made worse with movement. The patient rated her pain as ranging from 8 to 10 out of 10 on the numerical ranking scale (NRS). The patient still reported excruciating pain despite opioid escalation and the addition of several adjuvant medications. The chronic pain service was consulted due to difficulty in managing the patient’s pain, limiting the patient’s diet tolerance, and discharge planning. Upon evaluation, the patient was deemed a candidate for an erector spinae block. An ultrasound-guided technique was utilized by placing the patient in a prone position. A total of 20 mL of 0.25% bupivacaine was drawn up along with 40 mg of methylprednisolone. Under ultrasound guidance, the erector spinae approach to the paravertebral space was identified as superficial to the right T6 transverse process, and 10 cc of the anesthetic and steroid mixture were injected. The same process was repeated for the left T6 transverse process, and another 10 cc of the mixture was injected (Figure 1).

There were no complications of the procedure, and the patient reported complete relief of pain after the procedure. The patient did not require any additional pain medications for the remainder of her hospital course. The patient was discharged home the next day without a need for outpatient pain medication prescriptions.

## Discussion

Pancreatic innervation includes fibers that run through the celiac plexus and reach the lower thoracic segments of the spinal cord via the splanchnic nerve. These fibers are thought to stimulate visceral pain during inflammatory processes. When activated, action potentials travel along unmyelinated C-fibers and small myelinated Aδ-fibers of primary sensory neurons which synapse with secondary sensory neurons at laminae I, II, V, and X of the dorsal horn of the spinal cord at the T5-L2 level [2,3]. The patient in this case and others in recent literature have shown evidence that ESBs can provide relief in the visceral pain related to pancreatic inflammation by spreading cephalad and caudad through the paravertebral space, theoretically blocking dorsal and ventral rami of the spinal cord [4,5]. The spread of the drug to dorsal and ventral rami provides somatic anesthesia, and spread to epidural and paravertebral spaces provides visceral anesthesia. Steroids abolish afferent nerve conduction and increased the duration of action of ESB when combined with local anesthetics in nerve blocks [6]. Complications of the procedure include pleural puncture, local anesthetic toxicity, and post-block ecchymosis. These can be easily avoided with the use of ultrasound guidance to identify delineation of pleura and close monitoring of the patient after the procedure for complications [6].

This technique offers a superior alternative to traditional pain management strategies for acute chronic pancreatitis flares [3]. If performed in the emergency room setting, providing immediate and significant pain relief as evidenced in this case, there is potential for decreasing opioid consumption and hospital admissions related to these flares [4,6]. Furthermore, there is a potential benefit for more severe, intractable cases to place catheters for continuous infusions or intermittent boluses. This would subsequently increase patient satisfaction and reduce healthcare costs and resource utilization for the treatment of acute on chronic pancreatitis.

## Conclusions

ESBs are a safe and effective treatment option for visceral hyperalgesia related to acute on chronic pancreatitis and if employed earlier on in the course of the presentation, have the potential to significantly increase patient satisfaction and decrease healthcare utilization. In case of protracted or sub-acute course of acute pancreatitis, the ESB can be repeated or a catheter can be inserted at the level of the block for providing analgesia and to decrease the burden of oral analgesic medications.
